# Biodiversity and biogeography of the atmosphere

**DOI:** 10.1098/rstb.2010.0283

**Published:** 2010-11-27

**Authors:** Ann M. Womack, Brendan J. M. Bohannan, Jessica L. Green

**Affiliations:** Center for Ecology and Evolutionary Biology, 335 Pacific Hall, 5289 University of Oregon, Eugene, OR 97403-5289, USA

**Keywords:** biogeography, biodiversity, micro-organism, air, atmosphere

## Abstract

The variation of life has predominantly been studied on land and in water, but this focus is changing. There is a resurging interest in the distribution of life in the atmosphere and the processes that underlie patterns in this distribution. Here, we review our current state of knowledge about the biodiversity and biogeography of the atmosphere, with an emphasis on micro-organisms, the numerically dominant forms of aerial life. We present evidence to suggest that the atmosphere is a habitat for micro-organisms, and not purely a conduit for terrestrial and aquatic life. Building on a rich history of research in terrestrial and aquatic systems, we explore biodiversity patterns that are likely to play an important role in the emerging field of air biogeography. We discuss the possibility of a more unified understanding of the biosphere, one that links knowledge about biodiversity and biogeography in the lithosphere, hydrosphere and atmosphere.

*Noi viviamo sommersi nel fondo d'un pelago d'aria*. We live submerged at the bottom of an ocean of air.

(Evangelista Torricelli 1644 quoted in Middleton 1963)

## 1. Introduction

As humans, we have an intimate relationship with the air around us. This relationship is by and large unconscious; we breathe in without thinking, move through the eddies and tides of air often without notice. This largely unconscious relationship has led to a delayed appreciation of the air as a biological entity. But air is as alive as soil or water. Not only does it host large macroscopic organisms such as a soaring hawk or a drifting wildflower seed, but it also hosts a wide variety of micro-organisms. Hundreds of thousands of individual microbial cells can exist in a cubic metre of air (Burrows *et al.* 2009*b*), representing perhaps hundreds of unique taxa (Brodie *et al.* 2007; Fierer *et al.* 2008; Bowers *et al.* 2009). The ecology of these organisms—their diversity, distribution and interactions—is poorly understood. Given our intimate relationship with air, this lack of knowledge comes at a great cost. The life of the air, especially the microbial life, is in constant interaction with human life, both directly as a source of pathogenic and beneficial microbes (Kellogg & Griffin 2006) and indirectly through biological effects on atmospheric processes (Deguillaume *et al.* 2008). The atmosphere—the layers of air surrounding the Earth—has been described as ‘one of the last frontiers of biological exploration on Earth’ (Rothschild & Mancinelli 2001).

In this paper, we summarize our current state of knowledge of the ecology of the atmosphere, with an emphasis on the atmosphere's *biogeography*. Biogeography is the study of patterns in the distribution of life and the processes that underlie these patterns (Lomolino *et al.* 2006). The air has long been recognized as an important conduit for the movement of organisms from one geographical location to another, and thus is important for the biogeography of land and water. However, it is commonly assumed that the atmosphere is not a habitat in its own right but merely a conveyance for terrestrial and aquatic life. We review evidence that challenges this assumption and suggests the existence of metabolically active and actively reproducing organisms in the atmosphere. We argue that the atmosphere has a biogeography of its own. Our discussion will focus on micro-organisms, the numerically dominant forms of life in the atmosphere.

## 2. A brief history of aerobiology

Aerobiology has captivated scientists for centuries. Antoni van Leeuwenhoek—commonly known as the founder of microbiology—was one of the first to ask whether the air could be a habitat for micro-organisms (Gregory 1971), observing that ‘there may be living creatures in the air, which are so small as to escape our sight’ (van Leeuwenhoek 1941). Charles Darwin collected airborne dust on the HMS Beagle; this dust was found to contain 17 ‘different organic forms’ of micro-organisms (Darwin 1846). Micro-organisms have recently been isolated from these samples, demonstrating the ability of some airborne microbes to remain viable after long periods of time (Gorbushina *et al.* 2007). Darwin's contemporary Louis Pasteur, one of the first to systemically study airborne micro-organisms, showed that there are viable bacteria and moulds in the air, and that the densities of these organisms vary from location to location (Pasteur 1861).

When flight in fixed-wing aircraft became possible in the early 1900s, interest in aerobiology took wing as well. Phytopathlogist Fred C. Meier was perhaps the most enthusiastic proponent of studying microbes at high altitudes. Meier was adept at creating sampling devices and recruiting others to participate in his studies. Charles and Anne Lindbergh collected fungal spore samples for Meier using his ‘sky-hook’ device during a flight over the Arctic from North Haven, Maine to Copenhagen, Denmark (Meier & Lindbergh 1935). Amelia Earhart took Meier's collection device with her during her attempt to circumnavigate the globe. According to Meier, Earhart's collections, had she not perished during her voyage, would have been an ‘invaluable’ sample set that spanned the circumference of the globe over massive bodies of water where little sampling had been previously conducted (Montague 1937).

Despite this long and rich history of study, we know very little about the biology of the atmosphere relative to aquatic and terrestrial habitats. Technical limitations have hindered the study of the air. Low densities of micro-organisms in the air can make even sensitive molecular analysis difficult because of the small amount of biological material present in the air. Additionally, the lack of standardization in air collection and sample-processing methods complicate comparisons across studies (Kuske 2006; Peccia & Hernandez 2006). Owing to this lack of methodological standardization, it is unclear whether large differences in density estimates among studies can be attributed to biological variation (reviewed in Peccia & Hernandez 2006; Burrows *et al.* 2009*b*). Conceptual limitations also continue to impede the advancement of our understanding of life in the atmosphere. Most of what is known about airborne micro-organisms is based on the assumption that the atmosphere is a conduit for the dispersal of microbes rather than a dynamic habitat where micro-organisms actively metabolize and reproduce. Characterizing the role of biological processes in the atmosphere has enormous implications for furthering our understanding in a number of disciplines, from atmospheric chemistry and meteorology to biodiversity and biogeography.

## 3. An atmospheric habitat for micro-organisms

In the atmosphere, micro-organisms may belong to one of three groups—those that are not metabolically active, those that are metabolically active but rarely reproduce and those that are both metabolically active and actively reproducing. Microbes can form inactive propagules (e.g. spores) that disseminate through the atmosphere; however, for these organisms, the atmosphere would not be a ‘habitat’ in the conventional sense. We suggest that microbes that remain metabolically active in the atmosphere but rarely reproduce are organisms for which the atmosphere serves only as an accidental dispersal mechanism. The last group—both metabolically active and reproducing—can be thought of as ‘residents’ of the atmosphere. We argue below that, despite past assumptions, residents of the atmosphere are likely to exist, and that the atmosphere can act as a habitat for microbial life. We rely on four sources of information to make these arguments: that large portions of the atmosphere have environmental characteristics consistent with other microbial habitats; that biogeochemical cycling (probably mediated by microbes) occurs in the atmosphere; that at least some microbes found in the atmosphere are metabolically active; and that residence times of microbes in the atmosphere are long enough that actively reproducing residents could exist.

### (a) The atmosphere is not the most extreme microbial habitat

By several measures (pH, temperature, ultraviolet (UV) radiation, resource and water availability), the atmosphere appears to be less extreme than many other microbial habitats. The pH of clouds and rainwater ranges from 3 to 7 (Warneck 1988), a narrower range than that found in many microbial habitats. Microbes have adapted to a much wider range of pH conditions that occur in air, from highly acidic conditions near pH 0 (Schleper *et al.* 1995) to extremely alkaline conditions up to pH 11 (Jones *et al.* 1998).

Temperature can vary widely throughout the atmosphere, but includes ranges that are suitable for microbial life. In the lower atmosphere (up to 20 km above the Earth's surface), average temperatures decrease with altitude and range from an average of 15°C (at sea level) to −56°C (at 20 km) (NOAA NASA US Air Force 1976). Many micro-organisms are capable of growth at temperatures near and below 0°C (Morita 1975), with some communities reported to be metabolically active at temperatures as low as −18°C (Rothschild & Mancinelli 2001).

As with temperature, UV radiation, including DNA-damaging UVB, increases with altitude (Blumthaler *et al.* 1992). Increased UV radiation at higher altitudes does not necessarily mean that airborne micro-organisms are exposed to more UV radiation than their terrestrial counterparts, especially those terrestrial organisms that live at high elevations. Micro-organisms in the atmosphere may have a variety of methods for protection from UV radiation in addition to the suite of DNA-repair mechanisms found in all micro-organisms (Witkin 1976). It has been suggested that airborne microbes may mitigate levels of UV exposure by being embedded within larger particles with UV-attenuating properties, such as dust, pollen or water droplets (Lighthart 1997; Pearce *et al.* 2009). Pigments may also protect microbes from UV; the occurrence of pigmented micro-organisms in the atmosphere has been correlated with the presence of high levels of solar radiation (Tong & Lighthart 1997). These protective mechanisms are especially important for the survival of organisms at the upper level of the stratosphere, where levels of mutagenic UVB and UVC are not attenuated by the ozone layer (Smith *et al.* 1992).

Resource availability in the atmosphere is not necessarily lower than that of many terrestrial or aquatic environments. In clouds and rainwater, concentrations of nutrients (e.g. sulphate and nitrate) reach levels typical of oligotrophic lakes (Pearce *et al.* 2009). Numerous potential carbon sources are found in both clouds and the atmosphere, including carboxylic acids and alcohols (at concentrations up to 1 mg l^−1^; Pearce *et al.* 2009) as well as a variety of hydrocarbons (at concentrations up to 4 ng l^−1^; Warneck 1988). In addition to available resources for supporting heterotrophic metabolisms, the air provides a suitable habitat for phototrophs. Pigmented micro-organisms found in the atmosphere could be using pigments for photosynthesis. Gene sequences from putative photoautotrophs have been amplified from air samples (Brodie *et al.* 2007), although to our knowledge, no photoautotrophs have been isolated from the atmosphere.

### (b) Microbes in air are metabolically active

Direct *in situ* evidence of microbial metabolic activity in the atmosphere is rare and limited primarily to approaches that require culturing of microbes in the laboratory. For example, bacteria aerosolized in the laboratory have been shown to be capable of metabolizing glucose (Dimmick *et al.* 1975) and dividing (Dimmick *et al.* 1979), suggesting that aerosolization is not a barrier to metabolic activity and reproduction. Sattler *et al.* (2001) showed that micro-organisms incubated in cloud water at 0° have generation times of 3.6–19.5 days and take up labelled substrates at rates typical of bacteria in lake water. Micro-organisms isolated from cloud water degrade organic acids when cultured in artificial cloud water at 5°C and 17°C (Vaïtilingom *et al.* 2010).

These approaches have significant limitations. The environmental conditions microbes are exposed to in clouds (e.g. temperatures of −15°C in super-cooled droplets) cannot be easily reproduced in the laboratory. Culturing aerosolized microbes in the laboratory is likely to impose a bias and may not be representative of the airborne community (Pace 1997). A few studies have avoided these biases by using culture-independent methods for detecting metabolic activity. For example, Hill *et al*. (2007) observed that 76 per cent of cells in cloud water reduced the dye CTC (5-cyano-2,3-ditolyl tetrazolium), suggesting that this proportion was metabolically active. Measurements of ATP concentrations in cloud water approximate what would be expected for metabolically active cells at the cell density at which they are found in clouds (Amato *et al.* 2007*b*), suggesting that microbes can be metabolically active in the atmosphere.

### (c) Biogeochemical cycling may occur in the atmosphere

If metabolically active microbes are present in the atmosphere, they should leave chemical ‘footprints’ of their metabolisms. For example, microbes are intimately involved in biogeochemical transformations, and evidence for such transformations in the atmosphere would support the hypothesis of a resident microbiota. Nitrogen cycling in clouds (including mineralization and nitrification) has been demonstrated (Hill *et al.* 2007), suggesting the presence of metabolically active microbes. There is some evidence for carbon cycling in clouds, although it is not as clear-cut as the case for nitrogen. For example, bacteria have been isolated from clouds that are able to use organic compounds commonly found in cloud water, including acetate, formate, succinate, l-lactate, formaldehyde and methanol as carbon sources (Amato *et al.* 2007*a*; Vaïtilingom *et al.* 2010). Bacterial end products of these metabolic reactions are also commonly found in cloud water (Amato *et al.* 2007*a*), suggesting that these microbes are actively transforming these compounds in clouds. Microbial degradation of organic compounds in the atmosphere may not be limited to cloud aerosols. Bacteria have been collected outside of clouds that can degrade a variety of dicarboxylic acids, producing end products that can be further transformed in the atmosphere (Ariya *et al.* 2002).

### (d) Microbes likely go through multiple generations of growth in the atmosphere

The studies described above support the idea that the atmosphere is an environment capable of supporting resident (i.e. metabolically active and actively reproducing) microbial communities. The environmental stressors imposed by an aerial habitat are not unique, and there are multiple examples of micro-organisms that have adapted to live under conditions more extreme than those found in the atmosphere. If environmental conditions are not likely to prevent the presence of resident microbes in the atmosphere (at least in the lower atmosphere), what else might prevent their presence? It has been suggested (Sattler *et al.* 2001; Burrows *et al.* 2009*b*) that residence time may be the largest limiting factor for resident microbial communities in the atmosphere.

Residence times of microbes probably vary as a function of the size of the particles they are associated with, and as a function of air temperature and relative humidity, among other factors (Williams *et al.* 2002; Burrows *et al.* 2009*b*; Pearce *et al.* 2009). There have been no direct estimates of microbial residence times in the atmosphere. Currently, the best estimates of residence times are derived from mathematical models of particle transport (Williams *et al.* 2002; Burrows *et al.* 2009*a*). The most recent estimates of residence times for bacteria-sized particles range from 2.2 to 188.1 days (Burrows *et al.* 2009*a*). The shorter estimates assume efficient removal of bacteria by rain, ice and snow; the longer estimates do not assume this. Is this sufficient time for a resident microbiota to develop (i.e. to complete one or more generations)? Microbes have been shown to have generation times as short as 20 min, under ideal conditions, but under the conditions present in the atmosphere (cold and nutrient-poor), microbial generation times are likely to be substantially longer. As discussed above, Sattler *et al.* (2001) measured generation times of microbes in cloud water, and reported generation times of 3.6–19.5 days, similar to the generation times of microbes in cold, oligotrophic Arctic lakes (Panzenboeck *et al.* 2000). These rough estimates of residence and generation times suggest that at least some microbes could be undergoing more than 50 generations of growth while in the atmosphere.

## 4. What we know about air biogeography

Viewing the air as a microbial habitat has the potential to radically expand the scope of biodiversity and biogeography research. Biogeography has historically focused on understanding biological variation across the surface of the Earth, and has thus been primarily limited to the study of aquatic and terrestrial ecosystems. Understanding biological variation in aerial ecosystems opens the possibility for a truly unified view of biogeography, one that links biodiversity across each component of the biosphere: the lithosphere, hydrosphere and atmosphere. A preliminary picture of microbial life in aerial ecosystems is just beginning to emerge.

### (a) Density patterns for airborne microbes

The vast majority of aerobiology studies report patterns in the density (i.e. concentration) of micro-organisms (reviewed in Burrows *et al.* 2009*b*). Although the quantification of total, community-level abundance has a rich history in microbiology (Whitman *et al*. 1998), plant and animal surveys rarely report patterns in community-level abundance. This difference may reflect the reality that researchers commonly document what is most tractable to measure.

**Figure 1.**
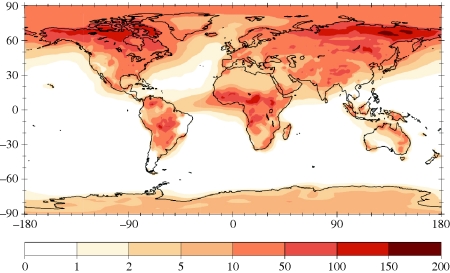
Simulated concentration (10^3^ m ^−3^) of 1 µm bacteria in near-surface air based on an adjusted general circulation model (Burrows *et al.* 2009*a*).

Aerobiologists have historically measured the density of culturable micro-organisms, reporting the number of colony-forming units per volume of air sampled (CFU m^−3^). Culture-based studies suggest that, as in terrestrial and aquatic systems, microbial densities vary with space, time and environmental conditions in the air. For example, the density of culturable microbes has been shown to decrease with increasing altitude (Fulton 1966), and numerous studies have documented seasonal and diurnal temporal variation in the density of culturable micro-organisms in the atmosphere (Bovallius *et al.* 1978; Lindemann & Upper 1985; Lighthart & Shaffer 1995; Tong & Lighthart 1999, 2000; Fang *et al.* 2007). Culture techniques, however, reveal only a fraction of microbial life. More recent studies use epifluorescent microscopy and report the total count of microbial cells per volume of air sampled (cells m^−3^). There is some evidence that total cell density counts from microscopy parallel culture-based counts (Tong & Lighthart 1999, 2000); however, few studies have enumerated airborne microbial densities using both approaches, making comparative inferences problematic. The density of micro-organisms in the atmosphere has also been estimated using particle transport models (Burrows *et al.* 2009*a*). Modelling approaches suggest that atmospheric cell density varies spatially, and that patterns in airborne cell density can occur on a global scale (figure 1).

### (b) Patterns of species distribution in the atmosphere

**Figure 2.**
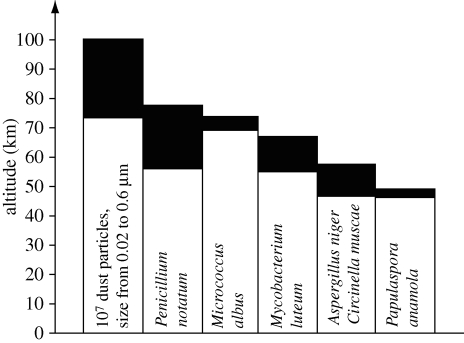
Isolation of microbes from the atmosphere. Shaded portions of the columns correspond to the altitude from which the organisms were sampled by a meteorological rocket and isolated in the laboratory. The first column depicts the altitude at which dust particles were sampled and detected (adapted from Imshenetsky *et al.* (1978)).

Although the majority of aerobiology has focused on community-level abundance patterns, culture-based research has provided a foundation for exploring taxa-level patterns. The study of taxa-level distributional patterns, such as a species' geographical range, is central to biogeography. Culture-based work has begun to address fundamental questions about the upper boundary of microbial geographical ranges in the atmosphere. Isolated cultures of the common mould, *Penicillium notatum*, have been collected at an altitude of 77 km, and the bacteria *Micrococcus albus* and *Mycobacterium luteum* at an altitude of 70 km (Imshenetsky *et al.* 1978; figure 2). Culture-based studies have also been used to understand the link between atmospheric environmental conditions and the occurrence of particular microbial species. For example, the occurrence of *Micrococcus* has been shown to correlate with the concentration of airborne particulate matter (Mancinelli & Shulls 1978); this might explain why airborne *Micrococcus* species are commonly dominant in urban environments (Fang *et al.* 2007). Finally, culture-based studies can help identify ubiquitous species that are likely to have large geographical range sizes. Spore-forming organisms, such as *Bacillus* species and other Gram-positives, tend to dominate culture-dependent surveys of airborne microbial diversity and thus may have large geographical ranges (Mancinelli & Shulls 1978; Lighthart 1997; Fang *et al.* 2007).

### (c) Airborne microbial community composition

Species do not exist in isolation, they occur together in complex ecological communities. To understand the mechanisms that shape biological variation on Earth, biogeographers study patterns in the composition and diversity of ecological communities in space and time. The development of environmental molecular biology has led to an explosion of investigations on the biodiversity and biogeography of microbial communities in terrestrial and aquatic environments (Green & Bohannan 2006). However, we currently know very little about microbial diversity in the atmosphere. Most studies demonstrating spatio-temporal variability of airborne microbial communities have been limited to culture-dependent methods. Recently, culture-independent molecular approaches have begun to be applied to airborne microbial communities. In contrast to culture-based studies, these molecular-based studies have revealed that airborne microbial assemblages can be as diverse as those in terrestrial environments, including soils (Maron *et al.* 2005; Brodie *et al.* 2007).

The first applications of molecular techniques in aerobiology typically analysed a single environmental sample (Hughes *et al.* 2004). More recently, investigators have begun to explore the biogeography of the atmosphere, by comparing microbial communities across multiple samples. Most comparative studies have focused on temporal variation in community structure at the same spatial location, with results ranging from pronounced differences in the daily (Fierer *et al.* 2008) and seasonal (Fröhlich-Nowoisky *et al.* 2009) cycles of airborne microbial community structure, to relatively static community structure across time (Bowers *et al.* 2009; Pearce *et al.* 2010).

There has been little research on the spatial variation of microbial communities in the atmosphere. Because microbial community composition can shift dramatically over short time scales (Fierer *et al.* 2008), comparative analyses between spatial locations require statistically controlling for time. Brodie *et al.* (2007) published the most definitive evidence for spatial variation of airborne microbial communities. In two Texas cities, these investigators pooled air filters in a manner that resulted in a random air sample per city, collected on a weekly basis for 17 weeks. In any given week, the data showed significant differences in community composition between cities; however, temporal and meteorological influences proved to be a greater factor in explaining variability of aerosol bacterial composition. Future research that explores spatial variability in microbial diversity, while accounting for temporal variability, will significantly expand our understanding of atmospheric biogeography.

## 5. Comparative biogeography of air, water and land

The studies reviewed above indicate that micro-organisms vary in abundance, distribution and diversity in the atmosphere. Yet, the air remains the least understood environment from a biogeographic perspective. Patterns in the variation of micro-organisms in the atmosphere have not been well documented, nor have the processes that underlie these patterns been identified. What might these patterns and processes look like in the atmosphere? Here, we consider defining attributes of land, water and air environments, and how these attributes may contribute to similar and different biogeography patterns across these domains. Building on a rich history of research in terrestrial and aquatic systems, we explore two patterns that are likely to play an important role in shaping the emerging field of air biogeography: environmental diversity gradients and the existence of biogeographic regions. Ultimately, a more unified understanding of the biosphere will entail comparing and contrasting these patterns across the lithosphere, hydrosphere and atmosphere.

### (a) An ocean of air

The vast majority of biogeographic studies to date have focused on terrestrial environments. However, there is increasing interest in the biogeography of marine environments (e.g. DeLong 2009; Tittensor *et al.* 2010), and marine biogeography may be the best model for what a biogeography of the atmosphere might look like. Landscape-scale analyses of terrestrial environments have often been reduced to two spatial dimensions (with soil depth ignored), simplifying both the measurement of biogeographic patterns and the development of theory to explain these patterns. But marine environments, much like the atmosphere, are unavoidably three dimensional. A given terrestrial environment (a particular forest, for example) is relatively long-lived and stationary; a given marine environment (e.g. a particular mass of ocean water) can be ephemeral and under constant motion, much like the atmosphere. We suggest that the major environmental gradients in marine environments (light/UV, temperature, nutrients etc.) vary in space and time at rates and scales more similar to the atmosphere than those of terrestrial systems. Given our assumption that atmospheric biogeography may be most similar to marine biogeography, we primarily focus our discussion below on the biogeographic patterns and processes shown to be important in marine systems.

### (b) Environmental diversity gradients in the atmosphere

Environmental gradients—geographical gradients in the abiotic and biotic environment—have been used for centuries as a tool to understand the ecological and evolutionary forces that shape biological diversity. Environmental gradients have inspired some of the earliest hypotheses about the origin and spread of life on Earth (Linnaeus 1781). Since Linnaeus, hundreds of studies of community structure along gradients of elevation, latitude and depth have contributed to the foundations of modern ecology and biogeography (Lomolino *et al.* 2006).

Despite the wealth of plant and animal environmental gradient research, there have been relatively few studies of microbial diversity gradients. The resounding message from recent microbial depth gradient research is that, as with macro-organisms, the structure and composition of microbial communities are significantly influenced by environmental variability. For example, in the ocean, temperature, pressure, light and nutrients vary from sea level to the sea floor. Recent culture-independent studies clearly demonstrate that this environmental variation influences the vertical distribution of oceanic microbial diversity, for example, patterns of taxonomic richness, RNA/DNA ratios, gene copy number and metabolic pathways (DeLong *et al.* 2006; Johnson *et al.* 2006; Wilms *et al.* 2006; De Corte *et al.* 2008; Brown *et al.* 2009; Treusch *et al.* 2009). In the atmosphere, temperature, pressure and moisture vary from sea level to the outermost layer of the atmosphere. Given the strong evidence for shifts in community structure along similar types of environmental gradients, it is parsimonious to assume that microbial biodiversity changes in predictable ways with altitude in aerial systems.

Another widely studied environmental diversity pattern is the increase in numbers of animal and plant species as one travels from the poles towards the tropics. This latitudinal diversity gradient has been recognized for centuries (Mittelbach *et al.* 2007), and in recent years, this pattern has received heightened attention in the microbial biogeography literature. Although the generality of latitudinal diversity gradients remains equivocal, both molecular-based studies (Fuhrman *et al.* 2008) and biodiversity models (Barton *et al.* 2010) have revealed a decrease in species richness with latitude for marine microbes. The most parsimonious explanation for an aerial latitudinal diversity gradient is that the diversity of the atmosphere reflects the diversity of terrestrial and marine systems (i.e. aerial communities are a random sample of metacommunities on the surface of the Earth). However, the possibility exists that the atmosphere has a unique latitudinal diversity pattern. Numerous mechanisms have been proposed to explain latitudinal diversity gradients that may be relevant in the atmosphere, including gradients in energy, temperature and moisture. To our knowledge, there have been no published studies on a latitudinal diversity gradient for micro-organisms in the atmosphere.

### (c) Biogeographic regions in the atmosphere

One of the most striking biogeographic patterns at a global scale is the existence of biogeographic regions. The globe is divided into six unique biogeographic regions, areas of the Earth's land surface that contain unique plants and animals (Wallace 1876; Lomolino *et al.* 2006). These unique biotas are hypothesized to exist because of vicariance, the evolutionary separation of species owing to historic barriers to dispersal. More recently, attempts have been made to define marine biogeographic regions (Lomolino *et al.* 2006). This is more challenging for several reasons: marine systems have fewer dispersal barriers, are more dynamic in space and time, have a more complicated geological history and are more obviously three dimensional in nature. Nonetheless, there are large-scale differences in marine biotas, even among pelagic organisms. These patterns are believed to be driven by environmental barriers (e.g. warm tropical oceans act as barriers to cold-adapted organisms) and differences in the biogeography of the underlying benthos and/or adjacent coastal regions, differences believed to reflect tectonic and oceanographic history.

**Figure 3.**
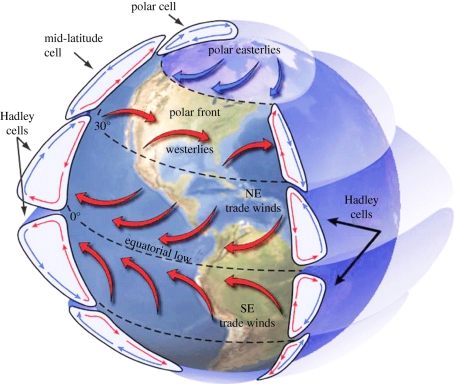
The six major air cells of the Earth's atmosphere (source: NASA).

Could there be biogeographic regions in the air? The short answer is that we do not know. No studies have attempted to ask whether there are large-scale patterns in the distribution of airborne micro-organisms. However, there are reasons to believe that such patterns are possible. There are large-scale patterns in the distribution of masses of air that could conceivably drive large-scale patterns in air organisms. At the largest scale, differential heating of air at the tropics and poles combines with the Earth's rotation to produce six ‘cells’ of air blanketing the globe (figure 3). Mixing of air is more frequent within cells than between them, resulting in barriers to air movement, and the potential for vicariance. These mixing barriers often coincide with strong differences in temperature between adjacent cells; thus, environmental barriers could augment physical barriers to dispersal. The major cells of air are relatively stable geographically, and rest on areas of the Earth's surface that are often biogeographically distinct. Thus the input of organisms to each cell could be distinct, and reflect tectonic and/or oceanographic history, much like the influence of benthic or coastal biogeography on marine pelagic distributions. Together, this suggests that large-scale patterns in the distribution of air organisms are possible. We suggest that a logical starting place for such studies is to ask whether airborne communities are more similar within the six major air cells than among them.

These major cells of air are restricted latitudinally (i.e. they occur within particular bands of latitude; figure 3). If these cells do represent biogeographic regions, differences in microbial community structure among these cells could reinforce latitudinal patterns in airborne microbial communities (if such patterns exist; see above). There is also the potential for biogeographic patterns within these major cells. Individual air masses (volumes of air with particular environmental characteristics) continually form, move and disperse within the major air cells. If these masses are sufficiently long-lived to allow for multiple generations of microbial growth, they could harbour unique resident microbial communities, analogous to patterns in the distribution of plant and animal communities in biogeographic ‘provinces’ within regions. However, to date, no study has attempted to determine whether microbial communities are more similar within particular air masses than among them.

## 6. Conclusion

Despite the potential importance of understanding the distribution of life in the air, there are major gaps in our current understanding of the air's biogeography. These gaps include a lack of accurate and comprehensive estimates of many important attributes of life in the air such as estimates of microbial densities and residence times, the proportion of organisms that are metabolically active, generation times of airborne organisms and the structure of airborne microbial communities. The use of new technology and standardization of techniques across studies will allow for a more complete understanding of the distribution of life in the atmosphere.

Most importantly, to move our understanding of life in the air forward, air biologists must learn to think like biogeographers. This includes designing studies that allow the disentangling of spatial and temporal effects on the distribution of life in the air, as well as using our knowledge of atmospheric dynamics to develop testable hypotheses regarding the biogeography of air. We feel that it would be especially fruitful to ask whether airborne communities are more similar within air cells and/or air masses than among them. Finally, studies of the biogeography of land and sea suggest that there are a number of biogeographic patterns that may be universal (Green & Bohannan 2006; Martiny *et al.* 2006). These include: (i) the distance–decay relationship (how similarity in community composition varies with the spatial, temporal or environmental distance that separates them), (ii) the taxa–area (or taxa–volume) relationship (how taxa richness increases with spatial scale), and (iii) latitudinal diversity patterns. These patterns are a promising starting point for developing a biogeography of the air.

The study of the biogeography of the air is in its infancy, but it has the potential to greatly alter how we think of the distribution of life on Earth. Given the important role the air plays in the dispersal of surface organisms, a more detailed understanding of the distribution of life in the atmosphere will allow us to better understand the distribution of life throughout the globe. It will also allow us to determine whether there are common patterns (and ultimately processes) underlying the distribution of life in the lithosphere, hydrosphere and atmosphere, bringing biologists a step closer to a comprehensive understanding of the distribution of all life.
